# Itaconic acid exerts anti-inflammatory and antibacterial effects via promoting pentose phosphate pathway to produce ROS

**DOI:** 10.1038/s41598-021-97352-x

**Published:** 2021-09-13

**Authors:** Xiaoyang Zhu, Yangyang Guo, Zhigang Liu, Jingyi Yang, Huiru Tang, Yulan Wang

**Affiliations:** 1 State Key Laboratory of Genetic Engineering, Zhongshan Hospital and School of Life Sciences, Laboratory of Metabonomics and Systems Biology, Human Phenome Institute, Fudan University, Shanghai, 200433 China; 2 CAS Key Laboratory of Magnetic Resonance in Biological Systems, State Key Laboratory of Magnetic Resonance and Atomic and Molecular Physics, Wuhan Institute of Physics and Mathematics, University of Chinese Academy of Sciences, Wuhan, 430071 China; 3 Wuhan Institute of Virology, The Chinese Academy of Sciences, Wuhan, 430071 China; 4 Singapore Phenome Center, Lee Kong Chian School of Medicine, Nanyang Technological University, Singapore, 636921 Singapore

**Keywords:** Bacteria, Bacterial host response, Adaptive immunity, Antimicrobial responses, Cytokines, Infection, Inflammation, Immunology, Microbiology

## Abstract

Itaconic acid is produced by immune responsive gene 1 (*IRG1*)-coded enzyme in activated macrophages and known to play an important role in metabolism and immunity. In this study, mechanism of itaconic acid functioning as an anti-inflammatory metabolite was investigated with molecular biology and immunology techniques, by employing *IRG1*-null (prepared with CRISPR) and wild-type macrophages. Experimental results showed that itaconic acid significantly promoted the pentose phosphate pathway (PPP), which subsequently led to significantly higher NADPH oxidase activity and more reactive oxygen species (ROS) production. ROS production increased the expression of anti-inflammatory gene A20, which in turn decreased the production of inflammatory cytokines IL-6, IL-1β and TNF-α. NF-κB, which can up-regulate A20, was also vital in controlling *IRG1* and itaconic acid involved immune-modulatory responses in LPS-stimulated macrophage in this study. In addition, itaconic acid inhibited the growth of *Salmonella typhimurium* in cell through increasing ROS production from NADPH oxidase and the hatching of *Schistosoma japonicum* eggs in vitro. In short, this study revealed an alternative mechanism by which itaconic acid acts as an anti-inflammatory metabolite and confirmed the inhibition of bacterial pathogens with itaconic acid via ROS in cell. These findings provide the basic knowledge for future biological applications of itaconic acid in anti-inflammation and related pathogens control.

## Introduction

Itaconic acid is an important mammalian metabolite which mediates the crosstalk between infection, immunity and metabolism. It is produced by immune responsive gene 1 (*IRG1*)-coded enzyme which catalyzes *cis*-aconitic acid in activated macrophages under conditions of infection or pro-inflammation^1–4^. The increased production of itaconic acid has been found in spleens of BALB/c mice infected with *Salmonella typhimurium*^5^. The *Schistosoma japonicum* worm and egg burden of mice co-infected with both *S. japonicum* and *S. typhimurium* were significantly reduced, and the elevated level of itaconic acid caused by *S. typhimurium* coinfection may play an important role^6^. In other bacterial infection studies, increased levels of itaconic acid in target organ or tissue have been found^7,8^. The itaconic acid has been shown to have an antimicrobial effect through inhibiting isocitrate lyase, the key enzyme of an essential pathway (glyoxylate shunt) for bacterial growth in vivo^9,10^. Itaconic acid can also inhibit bacterial and viral infections via modulating the production of reactive oxygen species (ROS)^11,12^, the function which is believed to belong to some of the antibiotics^13^.

Itaconic acid has demonstrated its anti-inflammatory functions in vitro and in vivo through the mechanisms which have been proposed in several investigations^14–18^. ROS play an important role in the anti-inflammatory mechanism of itaconic acid^15,16^, in which ROS has been shown to be generated from the fatty acid oxidation mediated by oxidative phosphorylation (OXPHOS)^11,16,19,20^. Endogenous itaconic acid may cause elimination of mitochondrial substrate-level phosphorylation in LPS-activated murine macrophage lineage^21^. Itaconic acid can inhibit succinate dehydrogenase, which may lead to alterations of tricarboxylic acid cycle (TCA) and modulate intracellular succinate level^14,22^. Itaconic acid also can resemble phenylpyruvate to suppress glycolysis by decreasing the level of fructose 2,6-bisphosphate (F26BP)^23^. Hence, more attention has been focused on glycolysis, TCA circle and OXPHOS when considering mechanisms of anti-inflammatory effects of itaconic acid^19,20,24^. There have been fewer studies on the pentose phosphate pathway (PPP), which is another glucose metabolic pathway in parallel to glycolysis^25^. PPP generates the pentose precursor for the synthesis of nucleotides and NADPH that may be catalyzed by NADPH oxidase to produce superoxide and other ROS^26,27^.

In the current investigation, an alternative mechanism of anti-inflammatory effects of itaconic acid with PPP involved was explored. *IRG1* in monocyte/macrophage-like cell line RAW264.7 was knocked out with CRISPR-Cas9 technique to prepare *IRG1*-null macrophage^28^, molecular biology and immune methods were employed to study how *IRG1* regulates macrophage responses to related stimuli through PPP pathway using this cell model. Experimental results demonstrated that itaconate can promote the pentose phosphate pathway to produce more NADPH, which was used by NADPH oxidase to produce more ROS. ROS also induced expression of gene A20 (*TNFAIP3*, tumor necrosis factor alpha-induced protein 3) and contributed to anti-inflammatory effects in RAW264.7. Significantly elevated ROS level inhibited the propagation of *S. typhimurium* in RAW264.7, and may be also the cause for the low hatching rate of *S. japonicum* eggs with presence of itaconic acid. Our research provided understanding into new mechanisms of the anti-inflammatory effects of itaconic acid/*IRG1* and shed new light on the anti-inflammatory functions of itaconate.

## Results

### Single cell selection of RAW264.7-IRG1KO

*IRG1* is the gene responsible for producing itaconic acid. In order to evaluate the function of itaconic acid, CRISPR-Cas9 was employed to delete the *IRG1* gene in RAW264.7 cell line. Twenty puromycin-selected positive single-cell clones were evaluated with Cruiser Enzyme kit. Gel-electrophoresis showed that single-cell clones (lane 2, 4, 7, 11) have the desired base mutations (Supplementary information: Fig. S1). In order to further confirm successful knock-out of *IRG1*, levels of itaconic acid in cells and cell culture medium was measured with NMR spectrometer. No itaconic acid was observed from the knock-out cell line and their spent medium irrespective of whether the cells were treated with lipopolysaccharide (LPS, 10 ng/mL) or not. However, the wild-type RAW264.7 was able to produce itaconic acid with or without LPS stimulation and more itaconic acid was produced with LPS treatment (Supplementary information: Fig. S2). This observation further confirmed successful deletion of the *IRG1* gene. The *IRG1* knocked out cell line was named as RAW264.7-IRG1KO.

### Endogenous itaconic acid attenuated LPS-induced inflammatory cytokines

**Figure 1:**
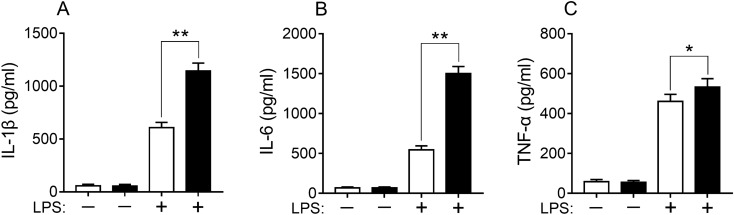
Different responses of RAW264.7 and RAW264.7-IRG1KO to LPS stimulation. ELISA kit test results of cytokines IL-1β (**A**), IL-6 (**B**) and TNF-α (**C**) in RAW264.7 and RAW264.7-IRG1KO cell culture medium when exposed to LPS (24 h). ITA, itaconic acid; LPS, lipopolysaccharide. White bar, RAW264.7; black bar, RAW264.7-IRG1KO. Values represent the mean ± S.E.M. *, *p* < 0.05; **, *p* < 0.01.

IL-1β, IL-6 and TNF-α are important pro-inflammatory cytokines which can be produced by activated macrophages. In this study, LPS significantly induced elevation in levels of IL-1β, IL-6 and TNF-α in both cell mediums of wild-type RAW264.7 and RAW264.7-IRG1KO cultured for 24 h when compared with control (Fig. 1A–C). Interestingly, immune response of *IRG1*-null cells to LPS treatment were more pronounced compared to its wild-type RAW264.7 cells (Fig. 1A–C), suggesting that endogenous itaconic acid was capable of diminishing LPS-induced inflammatory cytokines.

### Itaconic acid promoted the production of ROS that plays a role in diminishing LPS-induced inflammation

**Figure 2:**
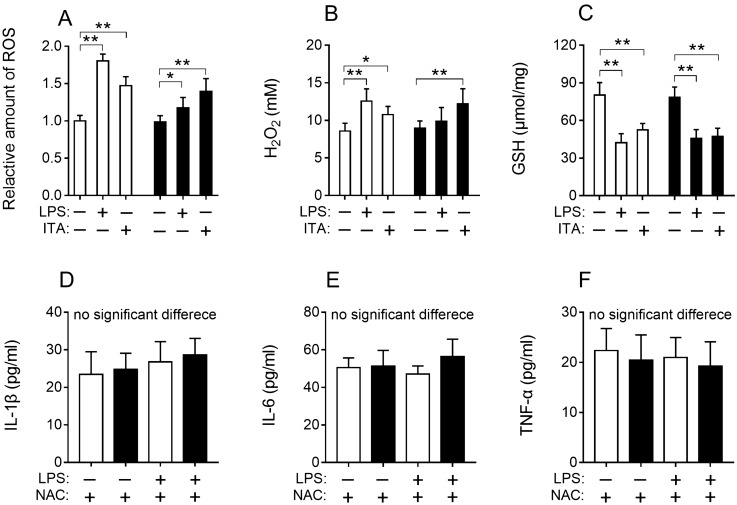
Results of ROS level in cell (**A**), H_2_O_2_ in cell medium (**B**) and GSH level (**C**) in cell lysate. The levels of IL-1β (**D**), IL-6(**E**), TNF-α (**F**) in cell culture medium from RAW264.7 and RAW264.7-IRG1KO with different treatments (24 h). White bar, RAW264.7; black bar, RAW264.7-IRG1KO. LPS, lipopolysaccharide; ITA, itaconic acid; NAC, *N*-acetyl-L-cysteine. Relative ROS amount in cells with different treatments was normalized to RAW264.7 control and represents the fold change. Values represent the mean ± S.E.M. *, *p* < 0.05; **, *p* < 0.01.

The levels of total ROS in cell, H_2_O_2_ in the cell culture medium and glutathione (GSH) in cell lysate were measured for cells treated with LPS or itaconic acid for 24 h. Experimental results showed that total ROS and H_2_O_2_ production in cell RAW264.7 subjected to LPS or itaconic acid was significantly increased compared to the corresponding untreated control (Fig. 2A, B). While the RAW264.7-IRG1KO cells produced significantly higher levels of H_2_O_2_ only when incubating with itaconic acid, and LPS treated RAW264.7-IRG1KO cells did not produce significantly increased level of H_2_O_2_ (Fig. 2B). These observations suggested that both endogenous and exogenous itaconic acid promoted the production of ROS and that the *IRG1* deletion could reduce the production of ROS. For RAW264.7-IRG1KO cells, treatment with itaconic acid also depleted level of GSH compared to untreated control cells, confirming that itaconic acid promoted the production of ROS (Fig. 2C). However, depleted levels of GSH were also observed in LPS treated RAW264.7-IRG1KO cells (Fig. 2C). *IRG1* can promote endotoxin tolerance via ROS in macrophage^16^. Therefore, ROS scavenger *N*-acetyl-L-cysteine (NAC, 2.5 mM) was added when treating RAW264.7 and RAW264.7-IRG1KO with LPS, and the results showed that no significant changes in the levels of IL-1β, IL-6 or TNF-α (Fig. 2D–F). This observation suggested that ROS scavenge could reduce LPS-induced inflammatory cytokines in both wild-type and RAW264.7-IRG1KO cells and eliminate the immune response difference between wild-type and RAW264.7-IRG1KO cells when treated with LPS. Therefore, knocking out of *IRG1* diminished the production of ROS in murine macrophage RAW264.7, and resulted in more pronounced pro-inflammatory cytokines production to LPS treatment when compared to wild-type RAW264.7 cells (Fig. 1A–C).

### Itaconic acid promoted the pentose phosphate pathway (PPP) and NADPH oxidase activity

**Figure 3:**
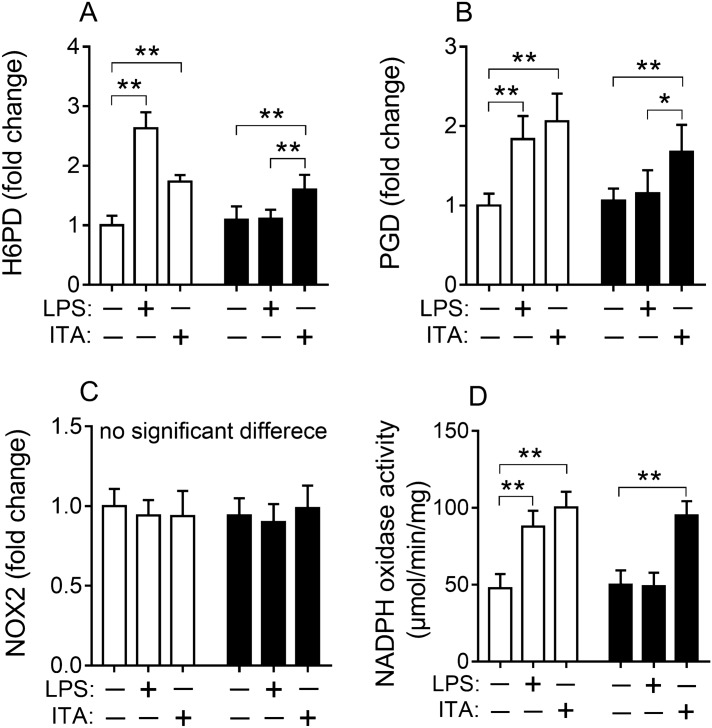
Relative expression of gene *H6PD* (**A**), *PGD* (**B**), *NOX2* (**C**) and NADPH oxidase activity (**D**) in RAW264.7 and RAW264.7-IRG1KO cells with different treatments (24 h). White bar, RAW264.7; black bar, RAW264.7-IRG1KO. LPS, lipopolysaccharide; ITA, itaconic acid. *H6PD*, glucose 6-phosphate dehydrogenase; *PGD*, 6-phosphogluconate dehydrogenase; *NOX2*, NADPH oxidase 2. Relative gene expression level of cells with different treatments was normalized to RAW264.7 control and represents the fold change. Values represent the mean ± S.E.M. *, *p* < 0.05; **, *p* < 0.01.

It is known that the first step of PPP pathway is the oxidative phase where NADPH is generated and can be used to produce ROS^29^. Hence, we hypothesized that higher production of ROS in the LPS treated wild-type RAW264.7 cells (compared with LPS treated RAW264.7-IRG1KO cells) may be generated from the PPP pathway. In order to test this hypothesis, the expression level of two key genes Glucose-6-phosphate dehydrogenase (*H6PD*) and 6-phosphogluconate dehydrogenase (*PGD*) in this pathway was tested. The expression levels of *H6PD* and *PGD* were significantly increased in the wild-type RAW264.7 cells treated with LPS for 24 h when compared to the control (Fig. 3A, B). However, this observation was not made for RAW264.7-IRG1KO cells with the same LPS treatment, given that there were no changes in the expression levels of *H6PD* and *PGD* was observed for RAW264.7-IRG1KO cells (Fig. 3A, B). Interestingly, the exogenous itaconic acid was also able to up-regulate the expression levels of *H6PD* and *PGD* for both wild-type and RAW264.7-IRG1KO cells (Fig. 3A, B), further confirming that itaconic acid stimulates PPP pathway. Gene (*NOX2*) expression level of NADPH oxidase was also tested, since this enzyme catalyzes the production of ROS using NADPH as substrate. However, no significant changes of *NOX2* expression was detected for both LPS and itaconic acid treated RAW264.7 and RAW264.7-IRG1KO cells (Fig. 3C). Then the enzymatic activity of NADPH oxidase in different treated cells was tested, and both LPS and itaconic acid treatments can upregulate the activity of NADPH oxidase in RAW264.7 cells (Fig. 3D), whilst only exogenous itaconic acid was able to promote the activity of NADPH oxidase in *IRG1*-null cells (Fig. 3D). Overall, these results suggested that itaconic acid promoted the PPP pathway, and up-regulated NADPH oxidase to produce ROS. While in the absent of itaconic acid, these effects were diminished.

### LPS and itaconic acid increased ROS in macrophages improved expression of gene A20 that was regulated by NF-κB pathway

**Figure 4:**
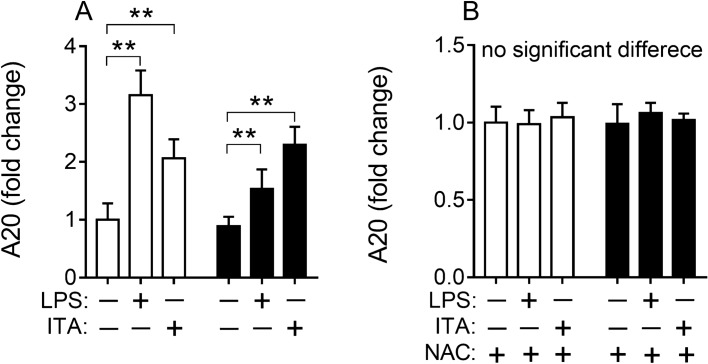
Relative expression of gene A20 in RAW264.7 and RAW264.7-IRG1KO cells with different treatments (24 h). White bar, RAW264.7; black bar, RAW264.7-IRG1KO. LPS, lipopolysaccharide; ITA, itaconic acid; NAC, *N*-acetyl-L-cysteine. A20, *TNFAIP3*, TNF alpha induced protein 3. Relative gene expression level of cells with different treatments was normalized to RAW264.7 control and represents the fold change. Values represent the mean ± S.E.M. **, *p* < 0.01.

Gene A20 (*TNFAIP3*, tumor necrosis factor alpha-induced protein 3) has been shown to be critical in restraining inflammation induced by endotoxin- and TNF-α-caused NF-κB responses^30,31^. The expression of A20 is up-regulated by ROS^15,16^. Therefore, A20 expression was investigated in wild-type RAW264.7 and RAW264.7-IRG1KO cells which were treated with LPS or itaconic acid. Both LPS and itaconic acid treatments induced higher A20 expression in both cell lines and with higher levels of A20 being expressed with the presence of endogenous itaconic acid (Fig. 4A). This effect was eliminated by *N*-acetyl-L-cysteine (NAC) treatment, an antioxidant capable of removing ROS, which is independent of *IRG1* regulation (Fig. 4B). NF-κB signaling pathway is considered a very important “rapid-acting” pro-inflammatory pathway^32,33^. Therefore, Bay11-7082 an inhibitor of NF-κB was employed to test the role of NF-κB in this study. The results showed that inhibiting NF-κB eliminated responses of A20 to LPS and itaconic acid treatments (Supplementary information: Fig. S3A). Additionally, when NF-κB was inhibited with Bay11-7082, the levels of IL-1β, IL-6 and TNF-α were unchanged when both wild-type RAW264.7 cells and RAW264.7-IRG1KO cells were treated with LPS and itaconic acid (Supplementary information: Fig. S3B–D). These observations confirmed that NF-κB, which can up-regulate A20, was vital in pro- and anti-inflammatory responses with *IRG1* or itaconic acid involved in LPS-stimulated macrophage.

### Endogenous and exogenous itaconic acid both can inhibit *S. typhimurium* replication in the macrophage

**Figure 5:**
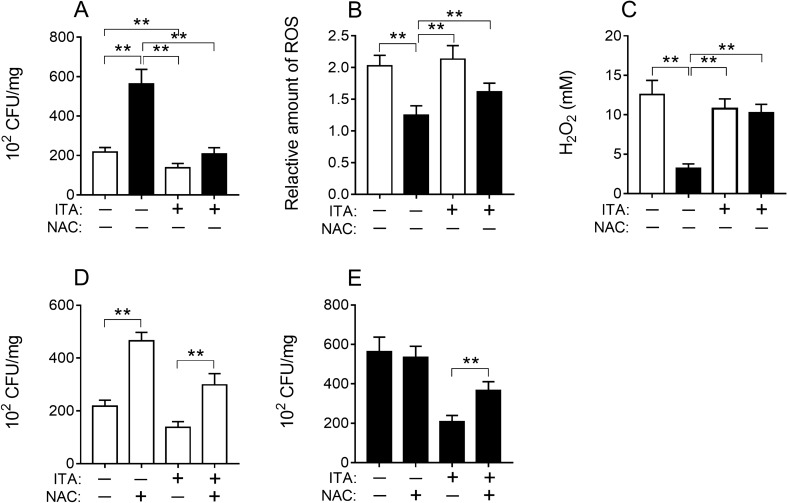
Growth of *S. typhimurium* in cells (**A**, **D**, **E**) and levels of ROS (**B**), H_2_O_2_ (**C**) in *S. typhimurium* infected cells and culture medium under different treatment conditions (24 h). White bar, RAW264.7; black bar, RAW264.7-IRG1KO. ITA, itaconic acid; NAC, *N*-acetyl-L-cysteine. Relative ROS amount in cells with different treatments was normalized to RAW264.7 control (NAC) and represents the fold change. Values represent the mean ± S.E.M. **, *p* < 0.01.

The macrophage plays an important role in the innate immune response to control infection^34^. Deletion of *IRG1* diminished the capacity of RAW264.7 to produce itaconic acid, and subsequently these cells were unable to suppress the growth of *S. typhimurium* (Fig. 5A). However, this capability was regained by adding itaconic acid exogenously; exogenous itaconic acid appeared to improve the capability of wild-type RAW264.7 in eliminating *S. typhimurium* further (Fig. 5A). Consistent with this finding, ROS levels produced by *IRG1*-null cells reduced significantly and adding exogenous itaconic acid could remedy some of this effect (Fig. 5B, C). Moreover, the effects of the removal of ROS with NAC on the capability of these cells in eradicating the *S. typhimurium* were further evaluated (Fig. 5D, E). The results showed that incubating these cells with NAC reduced ROS and the capability of eradicating the *S. typhimurium* (Fig. 5D, E).

### Itaconic acid inhibited the hatching of *S. japonicum* eggs

Previously, we have demonstrated that co-infection of mice with both *S. japonicum* and *S. typhimurium* can significantly reduce *S. japonicum* worm and egg burden^6^. Concurrently, we also discovered increased levels of itaconic acid in the spleen and liver of co-infected mice^5,6^. To test effects of itaconic acid on *S. japonicum* eggs, matured *S. japonicum* eggs from the liver of BALB/c mice after 5 weeks of *S. japonicum* infection were collected and subjected to different concentrations of itaconic acid. The result showed that even at a very low concentration, itaconic acid completely inhibited the hatching of *S. japonicum* eggs (Supplementary information: Fig. S4).

## Discussion

Itaconic acid has antimicrobial effect through inhibiting isocitrate lyase^9^ and has been considered not to be produced by mammalian cells for a long time^7^. However, this notion was subverted on the discovery that itaconic acid can be produced by immune-responsive gene 1 coded protein in LPS activated macrophages^2,3^. In addition, reactive oxygen species (ROS) has been known to play an important role in the anti-inflammation of itaconic acid^15,16^. Recently, the mechanism of the anti-inflammation of itaconic acid has been proposed through two ways: through IκBζ–ATF3 inflammatory axis regulated by electrophilic properties of itaconate and its derivatives^17^, or through activating Nrf2 via alkylation of KEAP^18^. These studies suggest that itaconic acid may be a potential anti-inflammatory drug^35,36^. In the current investigation, the mechanism of itaconic acid/*IRG1* regulating the pentose phosphate pathway to produce ROS was assessed for its anti-inflammatory and anti-bacterial activities.

As the first step of the investigation, an *IRG1*-null (RAW264.7-IRG1KO) cell line was generated by employing CRISPR-Cas9 to delete *IRG1* gene from the macrophage RAW264.7. Successful knockout of the *IRG1* gene was evident in the DNA and further manifested by the absence of endogenous production of itaconic acid (Supplementary information: Figs. S1 and S2). The RAW264.7-IRG1KO cells in the absence of itaconic acid had significant inflammatory response, e.g. increasing levels of IL-1β, IL-6 and TNF-α, when exposed to LPS (Fig. 1). This observed anti-inflammatory function of itaconic acid is consistent with studies conducted previously by other groups^37,38^. Immune-responsive gene 1 has been shown to be associated with ROS production^11,12,20^, therefore the role of ROS production in the anti-inflammatory functions of itaconic acid was subsequently tested in this study. The measurement results of ROS in cells, H_2_O_2_ in cell culture medium and GSH in cell lysates confirmed that significantly increased levels of ROS and decreased levels of GSH was associated with the presence of endogenous itaconic acid (Fig. 2A–C). In addition, when ROS in cells was removed by anti-oxidant (*N*-acetyl-L-cysteine) treatment, the anti-inflammatory effects of itaconic acid diminished in macrophages (Fig. 2D–F), which illustrated that anti-inflammation of itaconic acid could be mediated by ROS. Moreover, exogenous addition of itaconic acid could aid in the production of ROS (Fig. 2A, B). All the above results confirmed that anti-inflammatory effects of itaconic acid are via promoting the production of ROS, which is consistent with previously reported results showing that ROS functions as a signaling molecule^39^ and possesses anti-microbial activity^40–42^.

In order to identify where excessive amounts of ROS were generated, key enzymes in the pentose phosphate pathway (PPP) were investigated and the results showed that PPP was stimulated by the presence of itaconic acid. Since NADPH is the intermediate metabolite of the PPP and can be used by NADPH oxidase as a substrate to produce ROS, the expression levels of NOX2 and NADPH oxidase were subsequently evaluated. And it was found that both endogenous and exogenous itaconic acid can promote the PPP pathway and the activity of NADPH oxidase (Fig. 3A, B, D). The ubiquitin-editing enzyme A20 has been shown to regulate immune responses, including *IRG1*^16,43^. Thereafter the role of A20 in itaconic acid associated anti-inflammatory effects was subsequently assessed. Experimental results demonstrated that both exogenous and exogenous itaconic acid upregulated the expression of A20, and this upregulation could be eliminated by the antioxidant NAC (Fig. 4). Furthermore, inhibition of NF-κB by Bay11-7082 also can eliminate itaconic acid associated upregulation of A20 with concurrent eliminating anti-inflammatory effects of itaconic acid (Supplementary information: Fig. S3).

In this study, the experimental results showed that the anti-inflammatory effects of itaconic acid were motivated by its promotion of ROS production via PPP pathway, which is also regulated by A20 through the NF-κB pathway (Supplementary information: Fig. S3). And the increased levels of ROS induced by itaconic acid played an important role in inhibiting the growth of *S. typhimurium* in cell (Fig. 5). In addition, itaconic acid is found capable of suppressing parasitic growth, which is manifested by inhibiting hatching of *S. japonicum* eggs in vitro even at very low concentrations (Supplementary information: Fig. S4). These findings provided an rationale for our previous work, showing a reduction of schistosoma worm and egg burden in mice co-infected with *S. japonicum* and *S. typhimurium*, where the presence of itaconic acid was also concurrently discovered in the co-infected mice^6^.

**Figure 6:**
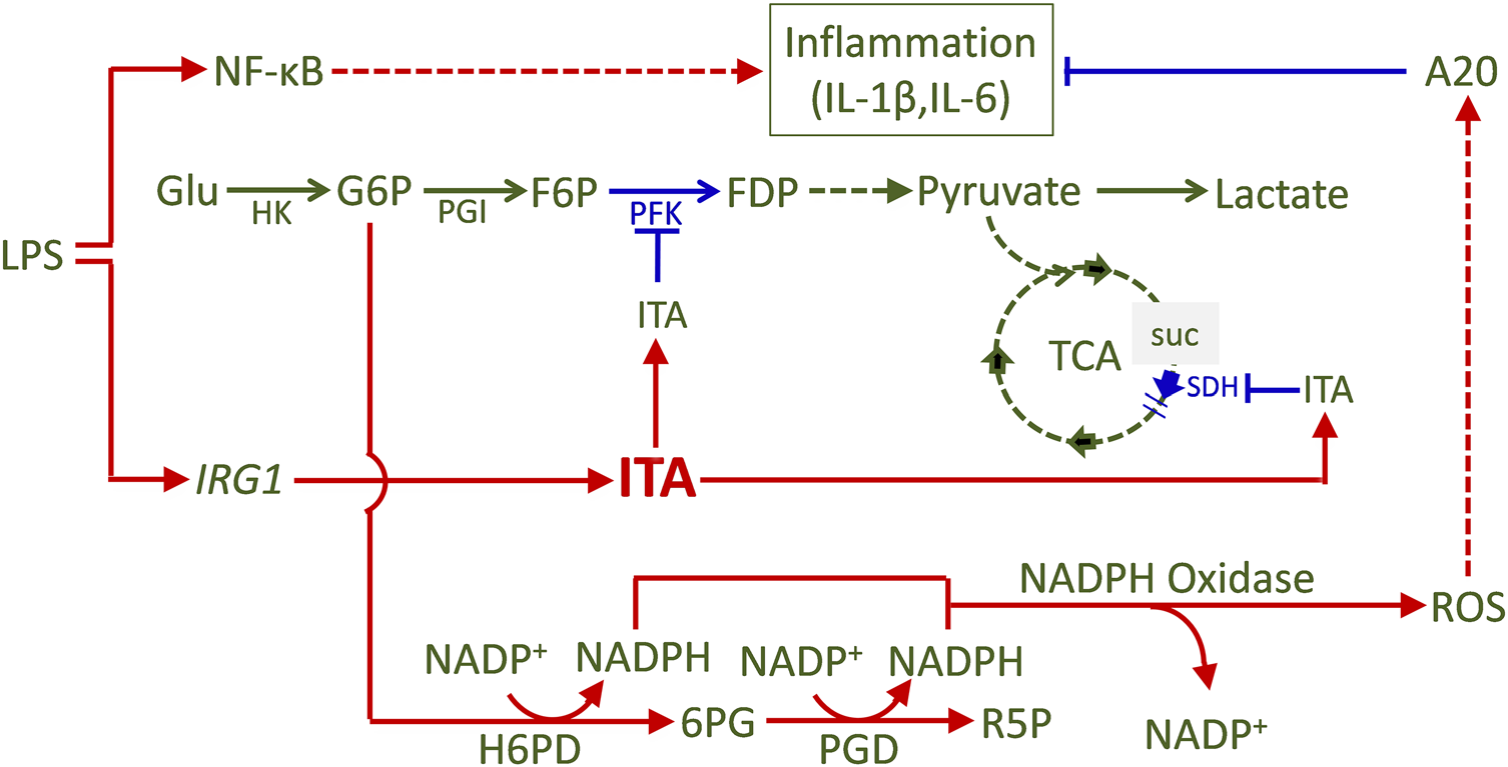
Schematic representation of the mechanism of anti-inflammatory effects of itaconic acid/*IRG1* (Drew with Microsoft Office Powerpoint 2007). Glu, glucose; HK, Hexokinase; G6P, glucose-6-phosphate; PGI, glucosephosphate isomerase; F6P, fructose-6-phosphate; PFK, phosphofructo kinase; FDP, fructose-1,6-diphosphat; H6PD, hexose-6-phosphate dehydrogenase; 6PG, 6-phosphogluconate; PGD, phosphogluconate dehydrogenase; R5P, ribulose-5-phosphate; ROS, reactive oxygen species; A20, TNFAIP3, TNF alpha induced protein 3; TCA, tricarboxylic acid cycle; SUC, succinate; ITA, itaconic acid; SDH, succinate dehydrogenase.

Itaconic acid, an under-studied small metabolite, has been found to play important functions in immune and metabolic regulation. The production of itaconic acid is controlled by the gene *IRG1*. The *IRG1*-null macrophage cell model, RAW264.7-IRG1KO, alongside its wild-type counterpart, RAW264.7, was employed to investigate the mechanism of anti-inflammatory effects of itaconic acid in this study. Itaconic acid promotes the production of ROS by stimulating the PPP-pathway. Intracellular ROS has the ability of eradicating cellular bacteria and can also induce the expression of anti-inflammatory gene A20 through NF-κB pathway, affecting the secretion of macrophage cytokines, and then exerting anti-inflammatory affects in vivo (Fig. 6). In addition, itaconic acid was showed having anti-parasitic effects. Our study has revealed an alternative mechanism on the anti-inflammatory functions of itaconic acid, in which metabolic pathway PPP involving ROS production play a necessary role.

## Methods

### *IRG1* knocked out in RAW264.7

RAW264.7 was cultured in DMEM medium with 10% fetal bovine serum at 37 ℃ with 5% CO_2_. CRISPR-Cas9 was carried out to knock out *IRG1* in RAW264.7 cells according to protocol published previously^44^. The plasmid, pSpCas9(BB)-2A-Puro (PX459)V2.0 (Addgene), was used to transfect RAW264.7 cells with FuGENE HD Transfection Reagent (Promega). The guide sequence was 5′-TGAGTGGCAGCGTTCGCTAT**GGG**-3′, and the synthetic oligonucleotide sequence need to be inserted in plasmid PX459 were forward: 5′-CACCGTGAGTGGCAGCGTTCGCTAT-3′ and reverse: 5′-AAACATAGCGAACGCTGCCACTCAC-3′. Single-cell clones were selected at 24 h after transfection and followed with puromycin (3 μg/mL, Sigma) treatment for 48 h. Primers used to amplify the target *IRG1* DNA fragment (~ 500 bp) from genome with possible indel were forward: 5′-TTGTCCTTCbTGGGCATGGATA-3′ and reverse: 5′-TCCAACAGCCAGATGTGAGAA-3′. This pair of primers was also used to amplify the target *IRG1* DNA fragment from wild-type RAW264.7 genome. Purified PCR products (~ 500 bp) of target *IRG1* DNA fragment from puromycin-selected single-cell clones and wild-type RAW264.7 cells were mixed together to produce hybrid-DNA fragments, and then the hybrid-DNA fragments were subjected to Cruiser Enzyme (Genloci Biotechnologies Inc.) validation. Cruiser Enzyme-digested hybrid DNA fragments were used to run DNA gel electrophoresis. PCR products from selected single-cell clones with indel in gene *IRG1* will have unpaired base when to produce hybrid-DNA fragment, which can be digested by Cruiser Enzyme to produce two different DNA fragment. In our experiments, it should produce a ~ 200 bp DNA fragment and a ~ 300 bp fragment. PCR products amplified from wild-type RAW264.7 were used as negative controls. The *IRG1* knocked out cell line was named as RAW264.7-IRG1KO, which was further confirmed for the absence of itaconic acid using nuclear magnetic resonance (NMR) spectrometer.

### Cell culture for gene expression, cytokines, ROS, H_2_O_2_, GSH and NADPH oxidase activity detection

Both wild-type RAW264.7 and RAW264.7-IRG1KO were cultured in DMEM with 10% fetal bovine serum at 37 °C with 5% CO_2_. Itaconate (10 mM, Sigma), lipopolysaccharide (LPS, 10 ng/mL, Sigma), *N*-acetyl-L-cysteine (NAC, 2.5 mM, Beyotime) and Bay11-7082 (1.5 μM, Beyotime) were used to treat cells and six repeats of each treatment were performed. Total RNA was isolated with RNAiso plus (Takara). RT-qPCR was carried out with PrimeScript RT Reagent Kit with gDNA Eraser (Takara) and SYBR Green PCR Master Mix (ABI). Relative expressions of genes *H6PD* (glucose 6-phosphate dehydrogenase), *PGD* (6-phosphogluconate dehydrogenase), *NOX2* (NADPH oxidase 2), A20 (*TNFAIP3*, TNF alpha induced protein) were measured using RT-qPCR and were normalized to expression of the housekeeping gene *β-actin*. Primers used for RT-qPCR analysis were listed in Table S1 (Supplementary information). IL-1β, IL-6 and TNF-α in cell culture medium were tested with ELISA Kits purchased from BOSTER (Wuhan, China). ROS in cell was tested with probe 2,7-dichlorofluorescin diacetate (DCFH-DA)-based Reactive Oxygen Species Assay Kit (E004-1–1, Nanjing Jiancheng Bioengineering Institute, China). H_2_O_2_ in cell culture medium (A064-1–1, Hydrogen Peroxide Assay Kit), glutathione (GSH) (A061-1–1, Glutathione Assay Kit) and NADPH oxidase activity (A127-1–1, NADPH Oxidase Assay Kit) in cell lysate were determined using respective kits purchased from Nanjing Jiancheng Bioengineering Institute (China).

### Infection of RAW264.7 and RAW264.7-IRG1KO with *S. typhimurium*

*Salmonella typhimurium* strain 14028 (ATCC) was obtained from Prof. Guo (Wuhan University) and cultured aerobically at 37 °C in Luria–Bertani (LB) broth (Sigma) overnight. Cultured bacteria were recovered by centrifuging at 10,000 g for 30 s, then washed twice and re-suspended in sterile PBS (pH7.2) with a suitable concentration. *Salmonella typhimurium* (~ 100:1) was added into medium of cultured cells (RAW264.7 and RAW264.7-IRG1KO). After 30 min of co-culture, gentamicin was added at a final concentration of 100 ug/mL to clear bacteria in medium. One hour later, medium was replaced with fresh medium containing 16 ug/mL gentamicin and maintained with or without treatments (10 mM itaconic acid, 2.5 mM *N*-acetyl-L-cysteine) for 24 h. Then cells were collected, weighted, and lysed for 10 min with tritonX-100 (0.1%). Cell lysates with bacteria were diluted with PBS into desired concentration and the bacteria number was determined by plate counts on solid LB culture medium. Six repeats were conducted for each of the treatment.

### Collection and itaconate treatment of *S. japonicum* eggs

*Schistosoma japonicum* cercariae were released from living oncomelanias^6^, which were purchased from Jiangsu Institute of Parasitic Diseases (China). Cercariae were counted in small dechlorinated water drops on cover slides under anatomical microscope. Six-week old SPF BALB/c mice (Hunan SJA Laboratory Animal Co., Ltd., China) were infected with approximate eighty *S. japonicum* cercariae each via abdominal skin. Forty five days after infection, mice were sacrificed and schistosome eggs were collected following the procedure: fresh liver tissues were cut into small pieces and homogenized and sequentially passed through nylon mesh sieves of 80, 120 and 300 mesh, washed with 0.9% saline to prevent eggs from hatching.

*Schistosoma japonicum* eggs were kept in 250 mL flask with dechlorinated water in artificial climate incubator at 26 ℃. Different concentrations of itaconic acid (0.00, 1.90, 3.80, 5.80, 7.70 μM) were used to treat *S. japonicum* eggs. Five repeats were conducted for each of the treatment. Then miracidia were counted under an anatomical microscope, 2, 4, 6 h after treatment.

### Ethics statement

All animal experiments were approved by the Animal Welfare Committee of Wuhan Institute of Physics and Mathematics, Chinese Academy of Sciences (Permission No. S-051–10-04-OU). All animal experiment procedures were carried out in strict accordance with the National Guidelines for Experimental Animal Welfare (People’s Republic of China, 2006), and in compliance with the ARRIVE guidelines.

### Statistical analysis

The data were subjected to either analysis of variance (ANOVA) followed by Tukey test for post hoc comparisons, and the software for statistical analysis used in this study is Prism 7.0 (GraphPad Software Inc., La Jolla, CA). Values represent the mean ± S.E.M.

## Supplementary Information

Supplementary Information 1.

## Data Availability

We declare that the data supporting the findings of this study are available within the article and its supplemental material files or from the corresponding author on request.
