# Supplementary material for: Itaconic acid exerts anti-inflammatory and antibacterial effects via promoting pentose phosphate pathway to produce ROS
Source: Sci Rep. 2021 Sep 13;11:18173. doi: 10.1038/s41598-021-97352-x (PMC8438069; doi:10.1038/s41598-021-97352-x)
Supplement: Supplementary file 1 — Supplementary Information 1. [file 41598_2021_97352_MOESM1_ESM.docx]

**Itaconic acid exerts anti inflammatory and antibacterial effects via promoting pentose phosphate pathway to produce ROS**

Xiaoyang Zhu^1^, Yangyang Guo^2^, Zhigang Liu^2^, Jingyi Yang^3^, Huiru Tang^1^, Yulan Wang^4,*^

^1^ State Key Laboratory of Genetic Engineering, Zhongshan Hospital and School of Life Sciences, Laboratory of Metabonomics and Systems Biology, Human Phenome Institute, Fudan University, Shanghai, 200433, China.

^2^ CAS Key Laboratory of Magnetic Resonance in Biological Systems, State Key Laboratory of Magnetic Resonance and Atomic and Molecular Physics, Wuhan Institute of Physics and Mathematics, University of Chinese Academy of Sciences, Wuhan 430071, China.

^3^ Wuhan Institute of Virology, the Chinese Academy of Sciences, Wuhan 430071, China.

^4^ Singapore Phenome Center, Lee Kong Chian School of Medicine, School of Biological Sciences, Nanyang Technological University, Singapore 636921, Singapore.

^*^ Corresponding author.

To whom the correspondences should be addressed: E-mail: [yulan.wang@ntu.edu.sg](mailto:yulan.wang@ntu.edu.sg); Fax: +65-65150417, Tel.: +65-69041106.

**Supplementary information**

**
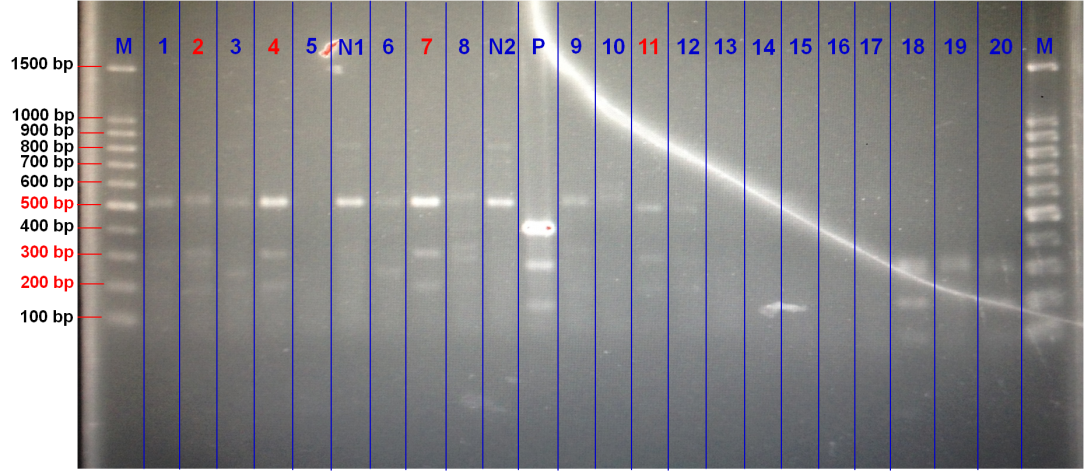
**

**Figure S1.** Gel-electrophoresis (2%) of Cruiser Enzyme-digested hybrid DNA fragments. M, 100 bp DNA ladder; lane 1-20, Cruiser Enzyme-digested hybrid DNA fragments samples; lane N1 and N2, Cruiser Enzyme-digested un-hybrid DNA fragment samples as negative control (purified PCR products from wild-type RAW264.7 cells); P, positive control. Lane 2, 4, 7, 11, show Cruiser Enzyme-digested positive result samples with a ~200 bp DNA fragment and a ~300 bp fragment.


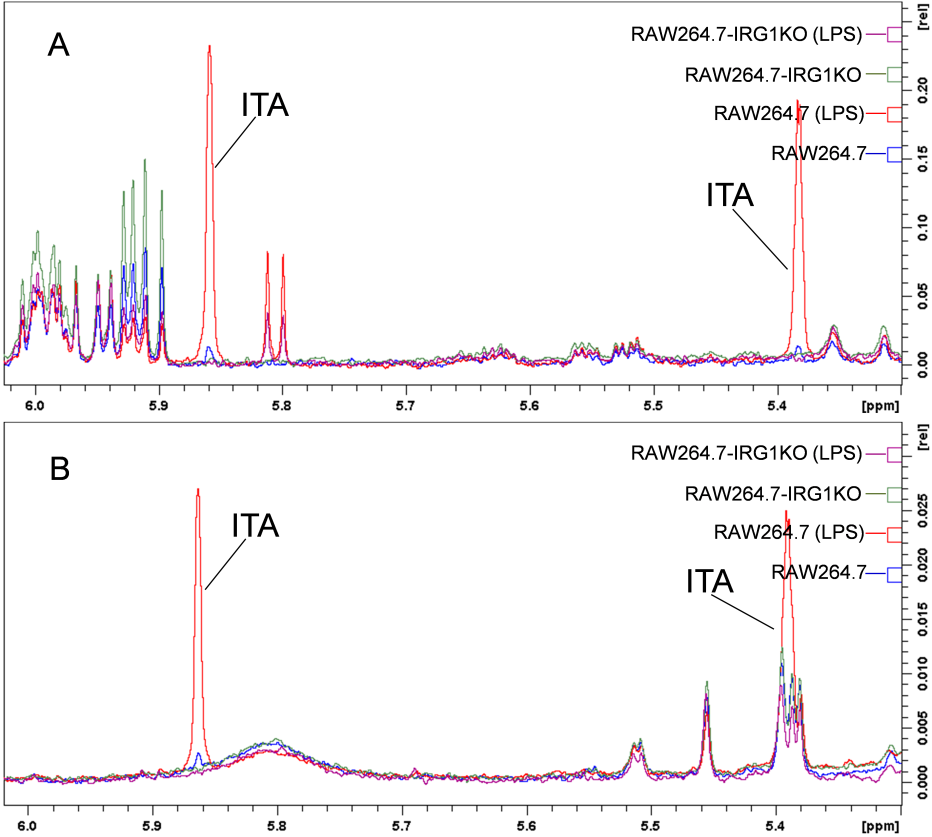


**Figure S2.** Different responses of RAW264.7 and RAW264.7-IRG1KO to LPS stimulation. Itaconic acid detection results with ^1^H NMR from sample of different-treated cell extracts (**A**) and corresponding cell culture medium (**B**). ITA, itaconic acid; LPS, lipopolysaccharide.


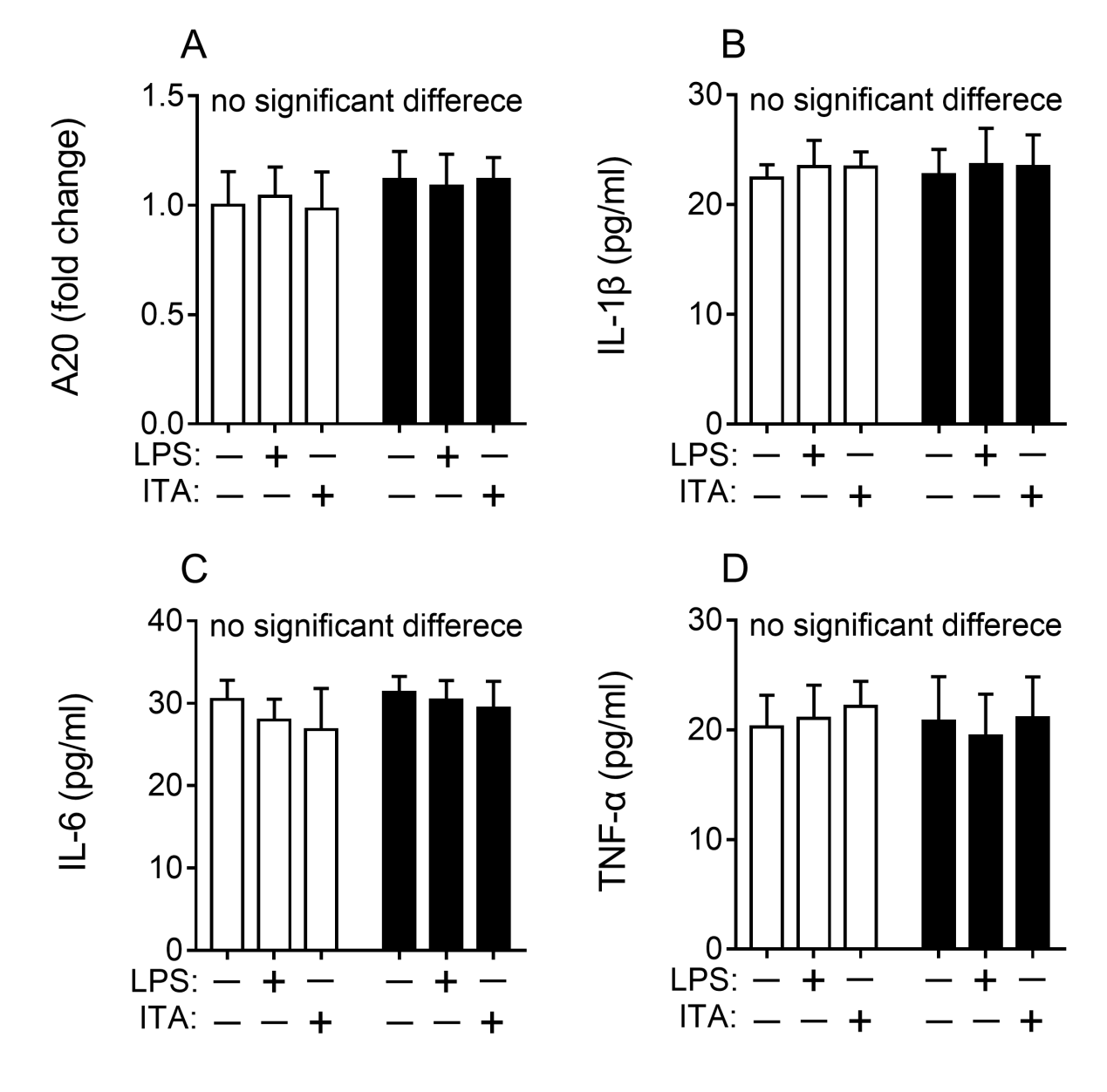


**Figure S3.** Expression levels of A20 (**A**) and concentrations of cytokine IL-1β (**B**), IL-6 (**C**) and TNF-α (**D**) in RAW264.7 and RAW264.7-IRG1KO with different treatments (24h). Cells in all groups were cultured with 1.5μM Bay11-7082, inhibitor of NF-κB. White bar, RAW264.7; black bar, RAW264.7-IRG1KO. LPS, lipopolysaccharide; ITA, itaconic acid. A20, TNFAIP3, TNF alpha induced protein 3. Relative gene expression level of cells with different treatments was normalized to RAW264.7 control and represents the fold change. Values represent the mean ± S.E.M. *, *p*<0.05; **, *p*<0.01. **
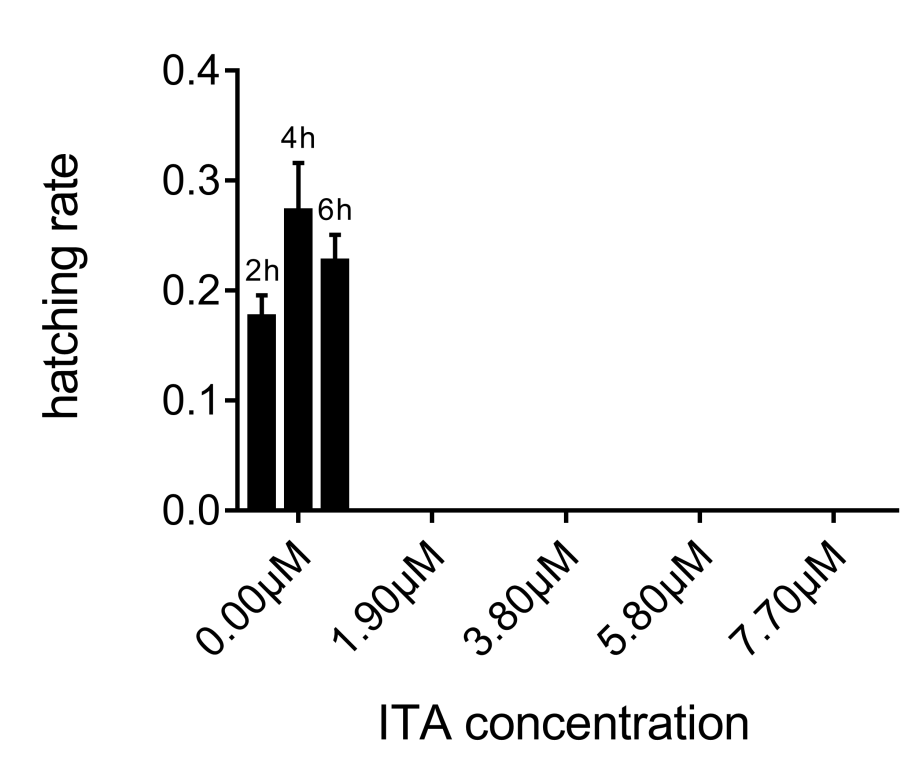
**

**Figure S4.** Hatch rate of Schistosoma japonicum eggs treated with different concentrations of itaconic acid for different durations (2, 4, 6 hours). Values represent the mean ± S.E.M.

**Table S1.** Primers used for RT-qPCR analysis.

| Target genes | Oligonucleotides | Sequences (5’ to 3’) |
| --- | --- | --- |
| β-actin | Forward primer | TGACAGGATGCAGAAGGAGA |
|  | Reverse primer | CGCTCAGGAGGAGCAATG |
| H6PD | Forward primer | ATGAAGCACACAGGCATTTGG |
|  | Reverse primer | TCCAGGTATAGCTGAAACAGTCC |
| PGD | Forward primer | TGAAGGGTCCTAAGGTGGTCC |
|  | Reverse primer | CCGCCATAATTGAGGGTCCAG |
| NOX2 | Forward primer | CCCTTTGGTACAGCCAGTGAAGAT |
|  | Reverse primer | CAATCCCGGCTCCCACTAACATCA |
| A20 | Forward primer | GCCTGTGCAAAAGAGATTTCAGAT |
|  | Reverse primer | TGATTCACAGAGCATGTAGGCC |
